# Urinary NT-proBNP: A Useful Biomarker for the Diagnosis of Respiratory Distress in the Neonatal Population

**DOI:** 10.7759/cureus.39019

**Published:** 2023-05-15

**Authors:** Evangelos Christou, Zoi Iliodromiti, Abraham Pouliakis, Eirini Loukatou, Pinelopi Varela, Adamantia Panagoulia, Anthia Chasiakou, Spyridon Zisimopoulos, Nicoletta Iacovidou, Theodora Boutsikou

**Affiliations:** 1 Department of Pediatrics, Panagiotis & Aglaia Kyriakou Children's Hospital, Athens, GRC; 2 Department of Neonatology, Aretaieion University Hospital, Athens, GRC; 3 Epidemiology and Public Health, 2nd Department of Pathology, National and Kapodistrian University of Athens, Athens, GRC; 4 Department of Pediatrics, Alexandra General Hospital, Athens, GRC; 5 Department of Biochemistry, Panagiotis & Aglaia Kyriakou Children's Hospital, Athens, GRC; 6 Department of Biopathology, Aretaieion University Hospital, Athens, GRC; 7 Department of Pediatrics, Elena Venizelou General and Maternity Hospital, Athens, GRC

**Keywords:** biomarker, urine, respiratory distress, #neonate, nt-pro bnp

## Abstract

Objective: To determine the diagnostic accuracy of urinary NT-proBNP levels in the detection and classification of the severity of respiratory distress in neonates after birth.

Methods: We compared the urinary NT- proBNP levels between the respiratory distress (RD) group and the control group on the 1st, 3rd, and 5th day of life (DOL).

Results: The RD group (55 neonates) showed higher levels of NT-proBNP compared to the control group (63 neonates) on DOL1 (585.4 pg/ml vs 396.1 pg/ml (p=0.014)), DOL3 (805.1 pg/ml vs 271.9 pg/ml (p<0.001)) and DOL5 (409.7 pg/ml vs 94.4 pg/ml (p<0.001)). Especially, on DOL5, the area under the ROC curve was 0.884 and the NT-proBNP cut-off value (221.8 pg/ml) showed a sensitivity of 71% and specificity of 79%. The RD group was subclassified into neonates with mild (21 neonates), moderate (19 neonates), and severe (15 neonates) disease. NT-proBNP cut-off point of 668 pg/ml for DOL5 can safely differentiate neonates with severe disease from those with mild and moderate disease (combined subgroups) since the sensitivity was 80% and specificity was 77.5% for DOL5.

Conclusion: Urinary NT-proBNP levels are a useful biomarker in detecting clinical signs of respiratory distress in neonates that are born within the first week of life; they can also detect neonates that are vulnerable to severe forms of the disease.

## Introduction

The function of the heart as part of the endocrine system of the human body was confirmed at least four decades ago with the discovery that it secretes two homologous natriuretic peptides - atrial natriuretic peptide (ANP) and brain natriuretic peptide (BNP) [1,2]. Today, it is known that cardiac myocytes in collaboration with cardiac fibroblasts are responsible for the production of all BNP-related peptides in the circulation system [3]. In the circulation system, the prohormone proBNP (108 amino acids) is separated into the biologically active hormone BNP (32 amino acids) by NT-proBNP (N-terminal part of the prohormone), mainly under the action of the proteolytic enzyme furin [4-5].

Even though the metabolic pathway of NT-proBNP has not yet been fully elucidated, its renal excretion is suggested as the main mechanism since it is detected in urine [6] and shows a good correlation with plasma creatinine concentration [7-8]. This is also supported by the fact that NT-proBNP levels are more affected by age than its BNP counterparts which are based on a gradual glomerular filtration rate (GFR) increase until two years of age [9]. Recent studies in neonates have shown an adequate correlation between NT-proBNP levels in blood serum and urine, as in the case of acute bronchiolitis [10], and hemodynamically significant patent ductus arteriosus (PDA) [11-12].

Respiratory distress in full-term neonates is caused by several underlying morbidities such as sepsis, pneumonia, transient tachypnea, meconium aspiration, and perinatal asphyxia [13]. In the last few years, major advances in perinatal care such as the administration of antenatal steroids, surfactant replacement therapy, newer modalities of assisted ventilation [14], nutritional support, and new biomarkers have led to a significant reduction in mortality and short-term morbidities in NICU neonates. NT-proBNP in serum and urine has already been studied in bronchopulmonary dysplasia (BPD); with relative safety, it can identify high-risk neonates and detect severe forms of the disease [15]. Therefore, its diagnostic accuracy in full-term neonates presenting with respiratory distress could also be investigated.

The present study aims to investigate a possible difference in urinary NT-proBNP levels between neonates with respiratory distress and the control group, on the 1st, 3rd, and 5th day of life (DOL1, DOL3, DOL5). Furthermore, we aim to establish biomarker values that have high sensitivity and specificity to distinguish neonates at risk of developing severe disease to provide early diagnosis and treatment.

## Materials and methods

Study design

The observational study was conducted from June 2020 to July 2022. We collected urine specimens from neonates at the Neonatal Department of Aretaieion Hospital (IN: 222/18-06-2020), while urine specimens from neonates with respiratory distress were collected at the NICU of Alexandra General Hospital (IN: 455/12-07-21) and Elena Venizelou Maternity Hospital (IN: 12124/5-6-20). Sample collection lasted from June 2020 to February 2022 and sample measurements and data analysis from March 2022 to October 2022. The study was conducted in accordance with the Strengthening the Reporting of Observational Studies in Epidemiology (STROBE) guidelines (Appendix 1) [16].

Setting

The study consists of two groups. The first group is referred to as the control group, in which neonates born to mothers who did not present any significant morbidity in pregnancy, with a gestational age >36 weeks postmenstrual age (PMA) were included. Neonates with perinatal morbidity such as hyperglycemia, in need of phototherapy for neonatal jaundice, of low birth weight, with IUGR, respiratory distress, Apgar score 1' <7, or in need of increased care at birth (for any reason) were excluded. The second group referred to as the “respiratory distress group" (RD group) included neonates with respiratory distress of any etiology after birth, and requiring additional treatment with O_2_. Exclusion criteria were gestational age <35 weeks PMA, birth weight <1500gr, presence of significant congenital anomalies, heart diseases affecting hemodynamic status, early-onset sepsis, and severe pathology not related to the respiratory system. Neonates of this group were classified based on the Silverman-Andersen respiratory severity score scale, which non-invasively classifies the severity of respiratory distress into mild, moderate, and severe. 

Variables

For all the neonates included in the study, demographic data for them and their mothers were collected through detailed recording of the data from the electronic database maintained in the respective hospitals. The NT-proBNP biomarker was measured in urine collected from the neonates on DOL1, DOL3, and DOL5, by placing a skin-friendly urine bag on the neonates. The diagnosis of respiratory distress for the neonates was based on the clinical findings of increased work of breathing such as tachypnea, nasal flaring, and chest retractions or grunting. If a neonate fulfilled one or more of these signs, they were classified as the RD group. As the biomarker is excreted through the kidneys, we checked the renal function via serum creatinine levels. In some cases, we monitored the 24-hour diuresis, as blood sampling is an invasive method for this age group. It is also known that NT-proBNP levels are particularly increased in congenital heart disease, therefore, in these neonates from the RD group where there was clinical suspicion for heart disease, a heart ultrasound was performed aiming at excluding them from the study [17].

Sample measurement

After urine samples were collected, they were frozen at -70°C in 2-3 ml aliquots. Before being measured, the samples were visually inspected for the presence of foreign bodies and if not completely clean, they were excluded from the measurements. They were then centrifuged at 10.000 rpm for 10 minutes. Samples were measured on the Architect i2000sr analyzer (Abbott Diagnostics, Abbott Park, IL, USA) (LoQ: 8.2 pg/ml, intra-assay CV: 2.8%, inter-assay CV: 3%, as stated by the manufacturer) at the Department of Biochemistry of Panagiotis & Aglaia Kyriakou Children's Hospital and at the Department of Biopathology of Aretaieion University Hospital by specialized laboratory staff. All samples were analyzed (using automated and commercially available standard methods) by performing regular internal and external quality-control procedures, in accordance with the national guidelines of good laboratory practice. The detection limits of the biomarker were 0 pg/ml - 35000 pg/ml. Sample values that were >35000 pg/ml in the initial measurement were further diluted to measure the exact values.

Statistical analysis

Based on our previous meta-analysis [18], we used an expected mean value for a healthy population - NT-proBNP level of 1341 pg/ml. We also estimated the standard deviation (based on the Cochrane Handbook) as 613 pg/ml. The required sample size for the two arms of the study (RD group and control group) was estimated using the G*power tool. We used a medium-to-large effect size (d=0.65), power of 1-β=0.95, and error probability of α=0.05. Other assumptions were equal populations of the RD group and control group and one-tailed tests since we know that NT-proBNP is expected to be higher in neonates with respiratory disease. The required sample size per group was 52 neonates. The statistical analysis was performed using the SAS software for Windows, version 9.4 (SAS Institute Inc., NC, USA) [19]. Descriptive values were expressed as median and quartile 1 (Q1) to quartile 3 (Q3) range and mean ±standard deviation (SD). Comparisons between the groups for the qualitative parameters were made using the chi-square test (and if required a Fisher exact test was performed). For the continuous parameters, normality was not possible to be ensured, therefore, non parametric tests were applied, specifically the Mann-Whitney U test and the Kruskal-Wallis test (if more than two groups). The significance level (p-value) was set to 0.05, thus the statistically significant difference between the compared groups was p<0.05.

## Results

A total of 118 neonates were included in the study in which urinary NT-proBNP was measured on DOL1, DOL3, and DOL5. Sixty-three of them belong to the control group and the remaining 55 to the RD group. A total of 145 neonates were recruited in the study, 70 and 75 in the control and RD groups, respectively. Four neonates were excluded from the control group because they developed respiratory disease in the first few days of life and three neonates because of the inability to collect the sample on the predetermined days. Seven neonates were excluded from the RD group because of missed antenatal diagnosis of congenital heart disease, four neonates because of death shortly after birth, seven neonates because of inability to collect the sample, and two neonates whose mothers ultimately refused to give consent to participate in the study despite their initial consent.

Baseline characteristics of the sample

The general characteristics of the study population are listed in Table 1. 

**Table 1:** Baseline characteristics between the study groups.

	RDS (N=55)	Control (N=63)		
Characteristic	Median (Q1-Q3)	Median (Q1-Q3)	p	OR and 95% CI
Gender female	26 (47.27%)	35 (55.56%)	0.460464	1.39 (0.67-2.88)
Gestational age at birth (weeks)	36w+2d (35w+3d – 37w+2d)	38w+5d (38w-39w+2d)	< .0001>	NA
Birth length (cm)	45 (44-47)	50 (48-52)	< .0001>	NA
Birth weight (g)	1980 (1650-2320)	3210 (2870-3500)	< .0001>	NA
1' Apgar score	8 (7-8)	9 (9-9)	< .0001>	NA
5' Apgar score	9 (8-9)	10 (9-10)	< .0001>	NA
Neonatal Blood Ph	7.3 (7.3-7.4)	7.3 (7.3-7.4)	0.06646	NA
Delivery				
Cesarean section	52 (94.54%)	35 (55.56%)	< .0001>	0.07 (0.02-0.26)
Vaginal delivery	3 (54.5%)	28 (44.44%)		
Maternal age	31 (27-35)	33 (30-37)	0.071603	NA
Parity	1 (1-2)	1 (1-2)	0.463986	NA
Assisted conception	8 (14.54%)	5 (7.94%)	0.377574	0.51 (0.16-1.65)
Mechanical ventilation	5 (9%)	0 (0.0%)	0.01446	NA
Maternal morbidity				
Any	33 (60%)	28 (44.44%)	0.1004	1.88 (0.9-3.9)
Diabetes	11 (20%)	10 (15.87%)	0.633043	1.33 (0.52-3.41)
Thyroid disease	12 (21.82%)	12 (19.05%)	0.819606	1.19 (0.48-2.91)
Other	17 (30.91%)	8 (12.70%)	0.0157	3.07 (1.20-7.85)
Prenatal steroids	35 (63.64%)	0 (0%)	< .0001>	NA
ΑΒΟ Incompatibility	10 (18.18%)	8 (12.7%)	0.450183	1.53 (0.56-4.19)
Rh Incompatibility	3 (5.46%)	3 (4.76%)	1	1.15 (0.22-5.97)

The screened variables include sex, gestational age, birth weight, birth length, 1' and 5' Apgar score, pH blood immediately after birth, type of delivery, maternal age, parity, type of conception (spontaneous or assisted), any coexisting maternal morbidity, administration of antenatal steroids, the need of mechanical ventilation, ABO and Rh Incompatibility. As expected, a shorter gestational age was observed in the RD group compared to the control group (p<0.0001), lower birth weight (p<0.0001), shorter birth length (p<0.0001), lower 1' and 5' Apgar score (p<0.0001). Furthermore, in the RD group, a predominance of cesarean section over vaginal delivery was recorded (p<0.0001), contrary to what was recorded for the control group. It is noteworthy that no statistically significant difference was recorded in the pH values between the two groups (7.3 vs 7.3, p=0.006).

ΝΤ-proBNP levels

**Figure 1:**
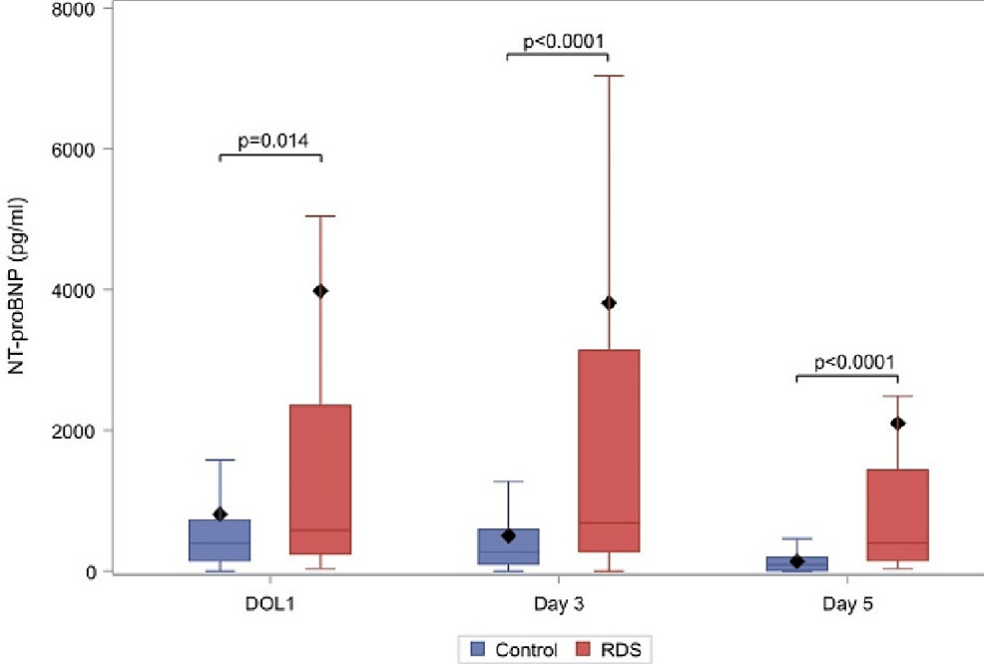
NT-proBNP levels for the respiratory distress group (N=55) and control group (N=63) on DOL1, DOL3, DOL5.

The levels of urine NT-proBNP per sampling day in the two groups are presented in Figure 1.

In Appendix 2, there is an analytical table of the results. For the RD group vs control group, higher values of the biomarker were recorded on all days but for DOL1 where the difference is not statistically significant (585.4 pg/ml vs 396.1 pg/ml, p=0.014). An important finding is also that biomarker values per neonate show a gradual decrease from DOL1 to DOL5 in both groups, despite the fact that in the RD group, the median value of NT-proBNP on DOL3 is higher than the corresponding median value on DOL1 (805.1 pg/ml vs 585.4 pg/ml). A least squares regression line based on the measurements per day was fitted, then we used the slope (alpha) of this line in order to determine the reduction rate (a negative alpha is indicative of reduction and a positive alpha for the increment of the NT-proBNP over time). The median reduction rate of the NT-proBNP for the RD group was -26.2 pg/ml per day (Q1-Q3: -431 pg/ml - -59 pg/ml) and the relative reduction rate for the control group was -53.4 pg/ml (Q1-Q3: -158pg/ml - -1.65 pg/ml), without a statistical significance (p=0.588). In the RD group and the control group, 63.4% and 72.5% of the neonates had decreased levels of NT-proBNP over time, respectively (p=0.29).

The diagnostic accuracy of NT-proBNP levels

**Figure 2:**
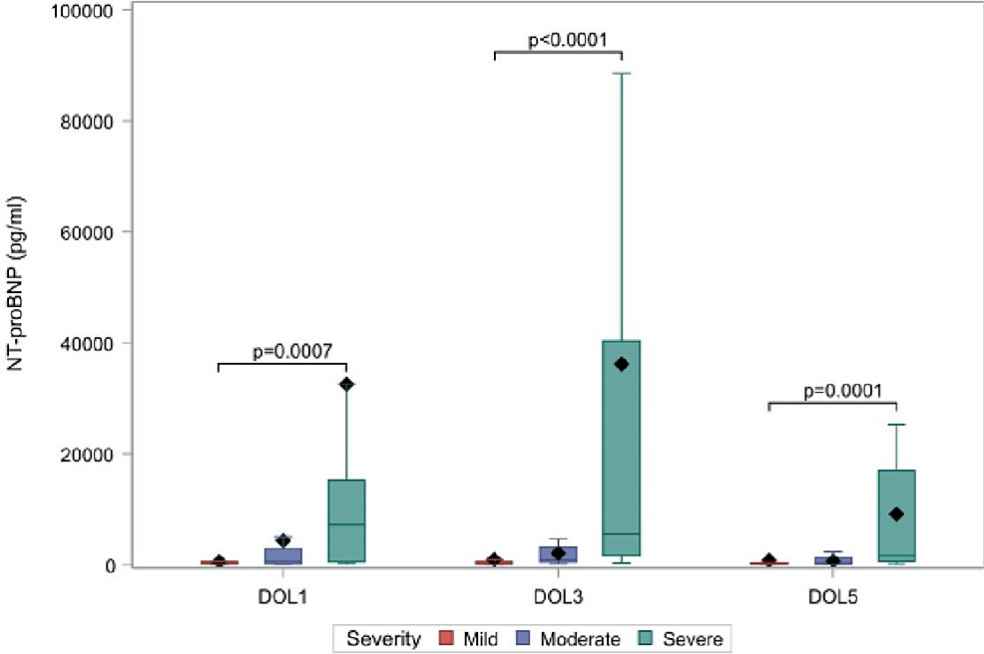
ΝΤ-proBNP levels between the mild (N=21), moderate (N=19) and severe (N=15) form of respiratory distress group (n=55) among the DOL1, DOL3, DOL5.

Initially, we used the respiratory severity score designed by Silverman-Andersen in 1956, which is a tool for the non-invasive assessment of the severity of respiratory distress in neonates. According to this, the RD group was further divided into subgroups with mild (21 neonates), moderate (19 neonates), and severe disease (15 neonates). NT-proBNP levels per sampling day in each subgroup are shown in Figure 2 and detailed in Appendix 3. We note that neonates who developed severe disease showed increased levels, compared to those with moderate or mild disease. In detail, on DOL1, it was 7187.4 pg/ml vs 668.4 pg/ml vs 299.8 (p=0.0007), on DOL3 5541.1 pg/ml vs 905.3 pg/ml vs 250.8 pg/ml (p<0.0001) and on DOL5 1655 pg/ml vs 448 pg/ml vs 221.8 pg/ml (p=0.0001). The gradual decline in the value of the biomarker in all groups over time is also recorded.

**Figure 3:**
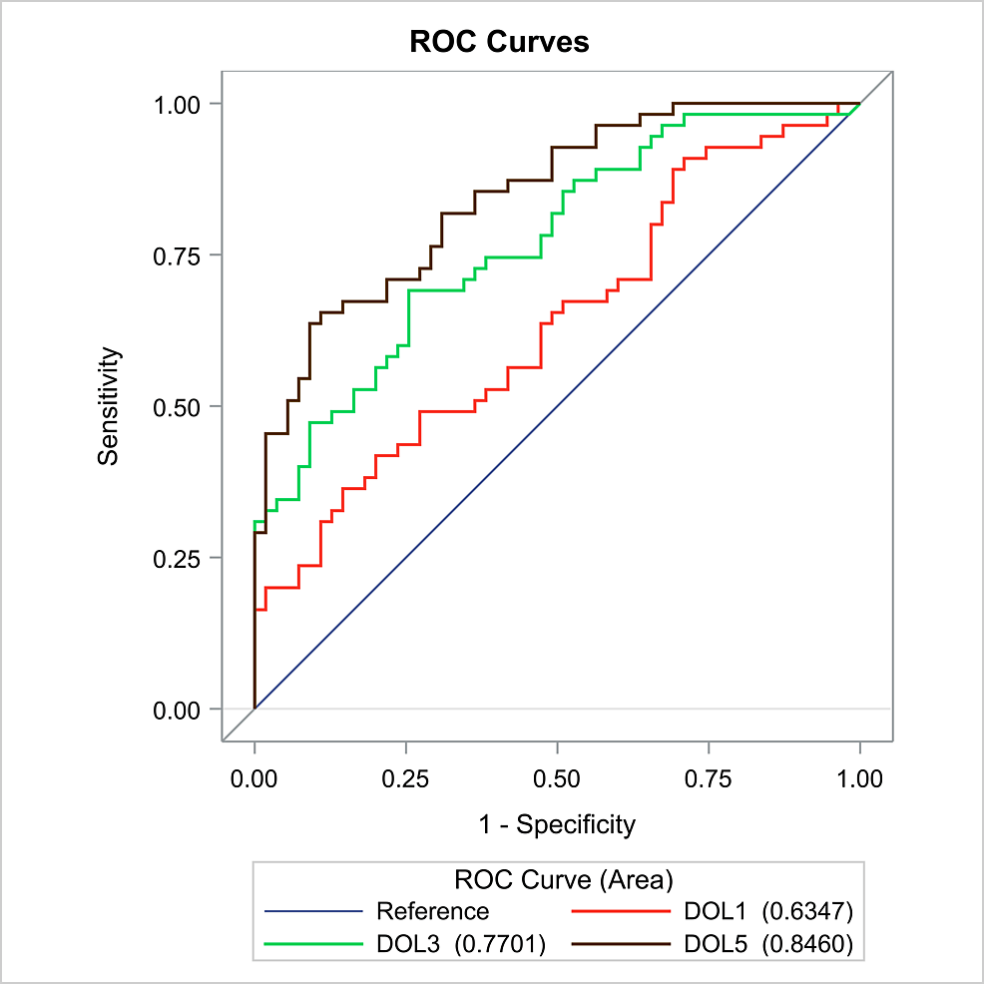
Receiver operating characteristic (ROC) curve of NT-proBNP for identifying respiratory distress among the DOL1, DOL3, DOL5.

We evaluated the discriminative power of the NT-proBNP in each individual day as a tool to separate the RD group from the control group. For this reason, we used all data (RD and controls) and created the ROC curves for the biomarker levels per day, for prediction of the respiratory distress. In detail, the ROC curves are shown in Figure 3. The AUC value of the biomarker was 0.63 (95% CI: 0.53-0.74) for DOL1, 0.77 (95% CI: 0.68-0.86) for DOL3 and 0.84 (95% CI: 0.78-0.92) for DOL5. There was a significant difference in the AUC of DOL1 vs DOL3 (p=0.007) but not between DOL3 vs DOL5 (p=0.1). On DOL3, when we chose the cut-off value of 458.3 pg/ml for the biomarker, a sensitivity and specificity of 69.1% and 70.5% were recorded, respectively, while for DOL5 at a cut-off value of 221.8 pg/ml, we observed an even greater sensitivity (70.9%) and specificity (79,3%). 

**Figure 4:**
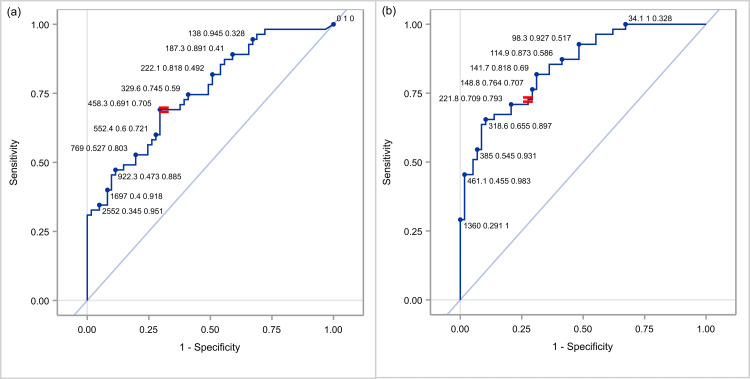
Various cut-off points of NT-proBNP for identifying respiratory distress at DOL3 (a) and DOL5 (b).

Figure 4 shows the sensitivity and specificity values for different NT-proBNP cut-off values (depending on the day). The difference in the optimal cut-off values arises as expected as a gradual decrease of NT-proBNP values from DOL1 to DOL5 has already been shown.

**Figure 5:**
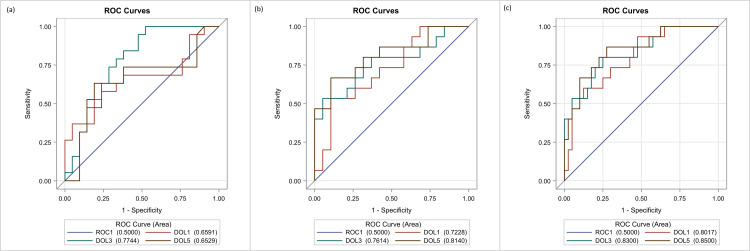
Receiver operating characteristic (ROC) curve of NT-proBNP between mild (a), moderate (b) and severe (c) form for identifying respiratory distress among the DOL1, DOL3 and DOL5.

Based on the fact that the levels of NT-proBNP are more elevated when the disease has higher severity, we evaluated the ROC curves for the discrimination of disease severity, per day. For these ROC curves were used only the data of the RD group. Since the ROC curves require a two-level approach, we evaluated the discrimination capability between the mild and the moderate disease, the moderate and the severe disease, and the severe and combined mild and moderate disease. The results are shown in detail in Figure 5 and in Appendix 4. Among others, we observed that the greatest performance is achieved on DOL5 between severe and combined mild and moderate disease, as the AUC value is 0.85. Similar but slightly smaller ROC curves are recorded for DOL1 and DOL3. 

**Figure 6:**
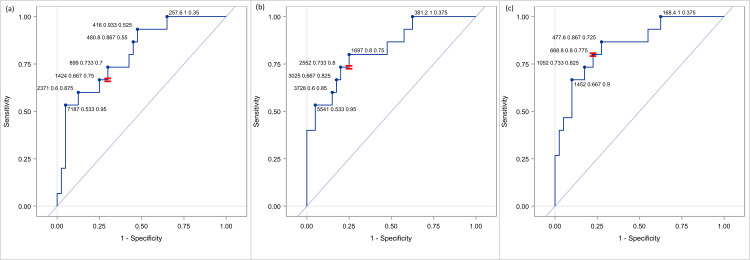
Various cut-off points of NT-proBNP for discrimination between severe form and combined (mild and moderate) form of the respiratory distress group at DOL1 (a), DOL3 (b) and DOL5 (c).

Based on the above, we attempted to find optimal cut-off points for discrimination between severe and combined mild and moderate type of the disease, per day (Figure 6). More specifically, when we set the threshold for DOL1 at 1,424 pg/ml, DOL3 at 2,552 pg/ml, and DOL5 at 668 pg/ml, the reliable positive and negative predictive value of the biomarker was confirmed, since it had a sensitivity of 66.7% and specificity 75% for DOL1, 73.3% and 80% for DOL3 and 80% and 77.5% for DOL5, respectively. 

## Discussion

In our study, the urine NT-proBNP levels in the RD group were higher than in the control group. Also, this biomarker helps detect severe forms of disease in neonates.

NT-proBNP values have a decreasing trend after birth, as the control group shows a median decrease of 53.4 pg/ml and the RD group a median decrease of 26.2 pg/ml, a finding that has previously been confirmed by other studies as well [20-22].

The gradual maturation of renal function, which is the basic clearance mechanism of the specific biomarker from the body, probably contributes decisively [23]. Also, the increase in peripheral resistance in contrast to the decrease in pulmonary vascular resistance and myocardial stress, which decreases day by day, plays a decisive role in the observed changes [24]. After the 1st week of life, NT-proBNP levels continue to decrease until the end of the 1st year of life but at a slower rate [23]. At the three time points, NT-proBNP levels were significantly increased in the RD group compared to the control group. Specifically, on DOL 1, the RD group vs control group was 585.4 pg/ml vs 396.1 pg/ml(p=0.014), on DOL 3 805.1 pg/ml vs 271.9 pg/ml (p<0.0001), and on DOL 5 409.7 pg/ml vs 94.4 pg/ml (p<0.0001). The biomarker has been investigated in several studies mainly in preterm neonates with RDS [25-26] and BPD [27-28] showing that it has increased diagnostic accuracy in the early detection of the diseases. In contrast, in neonates >35 gestational age only one study by Markovic-Sovtic [29] is available, in which results similar to ours are presented. In particular, when we set a cut-off value of NT-proBNP 458 pg/ml, on DOL3 it presented specificity and sensitivity of about 70% for both, while on DOL 5 with a cut-off value of 221.8 pg/ml, 70% and 80% respectively. It is observed that the biomarker has high positive and negative predictive value and could be used in daily clinical practice for early detection of neonates who may manifest respiratory distress and be treated appropriately.

Respiratory distress is a common disease in full-term neonates [30] while in late preterm neonates, it is 4.5 times higher [31] and is responsible for a series of short-term and long-term complications with adverse outcomes. Several times, the evaluation of respiratory distress is demanding. Among various invasive or non-diagnostic scores designed for its evaluation, the respiratory severe score designed by Silverman-Andersen is a non-invasive scale with high diagnostic accuracy and is relatively easy to apply [32]. Based on this, neonates in the RD group were classified into neonates with mild, moderate, and severe disease. On all days, neonates with severe disease recorded much higher levels of the biomarker than the other two subgroups. More specifically, at NT-proBNP cut-off levels on DOL1 1424 pg/ml, DOL3 2552 pg/ml, and DOL5 668 pg/ml, the group with severe disease could be separated from the combined group of neonates with mild and moderate disease with sensitivity and specificity between 65% and 80%, on all days.

The choice to measure the biomarker in urine rather than in blood serum in neonates was based on the fact that it is a non-invasive, safe, and validated method of measurement. In adults, it was reported that it shows an excellent correlation with its levels in the blood serum [33] as a result of which it is used in daily clinical practice for the diagnosis and monitoring of diseases such as heart and kidney disease [34], left ventricular hypertrophy [35], acute coronary syndrome [36] and others. Also, the cost of measurement in the urine is equal to the blood. A key finding is that the use of urine NT-proBNP testing to support respiratory distress diagnosis is likely to improve diagnostic accuracy in true-positive and true-negative cases, as well as reduce serious adverse events and diagnostic workups. So, the use of NT-proBNP as a diagnostic tool can improve the clinical assessment and management of high-risk neonates, resulting in improved patient-relevant health outcomes, and is likely to be a cost-saving option. 

NT-proBNP is a sensitive biomarker in the assessment of cardiopulmonology status and diseases of neonates. Due to the nature of respiratory distress in late preterm, early term, and term neonates, as a progressive, multifactorial, and no predictable disease, it is necessary to use a simple, fast and easy procedure in daily clinical practice to make decisions. The early interventions regarding the mode of ventilation, the supportive therapy, and the de-escalation of the therapy prevent the long-term morbidity. The serial measurements of the urinary NT-proBNP in the term neonates who are born with symptoms of respiratory distress within the first week of life or in the high-risk term neonates, the evaluation of the levels variance, and the establishment of reliable cut-off values could be significantly helpful.

Although the results of the present study are interesting and offer important findings for the usefulness of the biomarker in neonates with respiratory distress, they need to be confirmed by more studies as there are some limitations. First, because of the nature of the study, even though there were no significant differences between the two groups, there is probably an inherent risk of bias because of the failure to measure potential confounders that characterizes all prospective observational studies. Second, the study sample, although satisfactory, remains small and the results should be investigated in a larger neonatal population. Finally, although a non-invasive scale was used to categorize the severity of respiratory distress, it has limitations in terms of its diagnostic accuracy.

## Conclusions

Urinary NT-proBNP is potentially an accurate diagnostic biomarker for detecting respiratory distress in neonates' first days of life. It also has high sensitivity and specificity in diagnosing neonates who might develop severe disease, thereby contributing to their early treatment and a more favorable clinical outcome. In the future, multicenter studies need to be carried out before the biomarker is established in daily clinical practice and becomes a reliable tool in the management of a common pathology that occurs in full-term neonates.

## Figures and Tables

**Figure 7:**
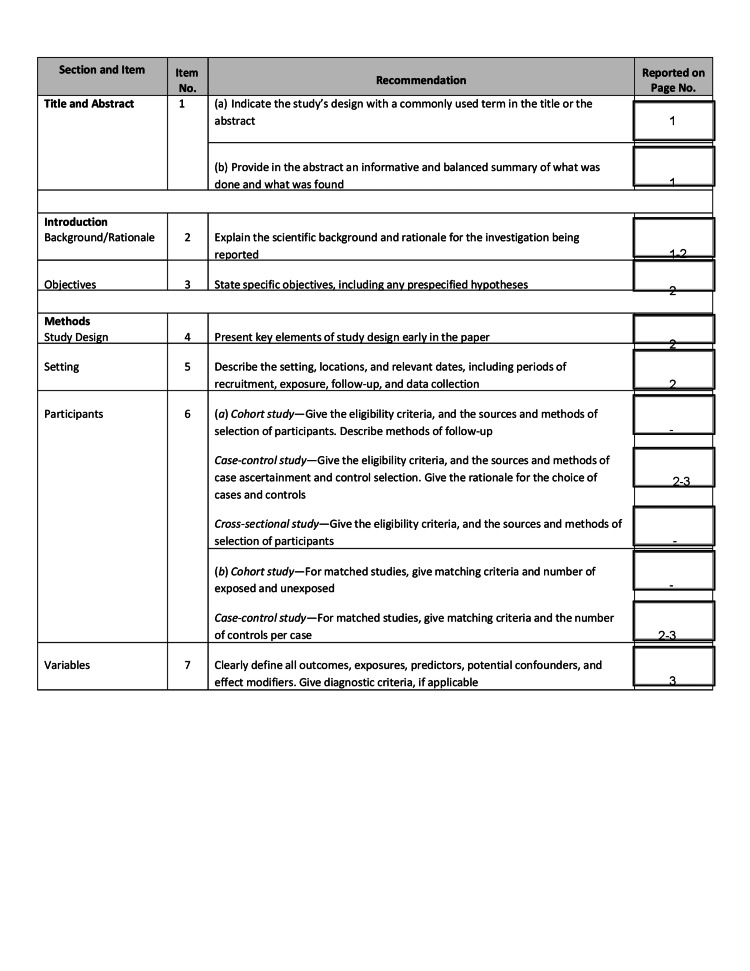
STROBE guideline checklist

## Appendices

Appendix 1

Appendix 2

**Table 2:** NT-proBNP levels between the RD (respiratory group) group and control group among the DOL1, DOL3, DOL5.

NT-proBNP levels	RDS (N=55)	Control (N=63)	
	Median (Q1-Q3)	Median (Q1-Q3)	p
NT-proBNP day 1 (pg/ml)	585.4 (241.4-2371.2)	396.1 (147.2-726.9)	0.0142
NT-proBNP day 3 (pg/ml)	805.1 (280.4-3878.1)	271.9 (101.3-588.2)	< .0001
NT-proBNP day 5 (pg/ml)	409.7 (148.8-1452.1)	94.4 (7.3-196.8)	< .0001

Appendix 3

**Table 3:** ΝΤ-proBNP levels between the mild, moderate and severe form of RD group among the DOL1, DOL3, DOL5.

NT-proBNP levels	Mild (N=21)	Moderate (N=19)	Severe (N=15)	
	Median (Q1-Q3)	Median (Q1-Q3)	Median (Q1-Q3)	p
NT-proBNP day 1 (pg/ml)	299.8 (200-585.4)	668.4 (186.4-2907.3)	7187.4 (571.2-15268)	0.0007
NT-proBNP day 3 (pg/ml)	250.8 (147.9-600.8)	905.3 (539.5-3238.7)	5541.1 (1696.9-40354)	< .0001
NT-proBNP day 5 (pg/ml)	221.8 (141.7-366.7)	448 (114.9-1276.8)	1655 (668.8-16989)	< .0001

Appendix 4

**Table 4:** Receiver operating characteristic (ROC) curve of NT-proBNP between the mild (a), moderate (b) and severe (c) form for identifying respiratory distress at DOL1, DOL3 and DOL5.

Comparison of the groups	NT-proBNP day	AUC	Standard Error
Moderate from mild	Day 1	65.91% (47.97% - 83.86%)	9.16%
	Day 3	77.44% (62.61% - 92.28%)	7.57%
	Day 5	65.29% (46.74% - 83.84%)	9.46%
Severe from moderate	Day 1	72.28% (54.8% - 89.76%)	8.92%
	Day 3	76.14% (58.81% - 93.47%)	8.84%
	Day 5	81.4% (66.25% - 96.56%)	7.73%
Severe from combined mild and moderate	Day 1	80.17% (67.34% - 93%)	6.55%
	Day 3	83% (70.55% - 95.45%)	6.35%
	Day 5	85% (73.48% - 96.52%)	5.88%43
